# The relationship of hyperlipidemia with maternal and neonatal outcomes in pregnancy: A cross-sectional study

**DOI:** 10.18502/ijrm.v17i10.5294

**Published:** 2019-11-07

**Authors:** Seyedeh Hajar Sharami, Zahra Abbasi Ranjbar, Fatemeh Alizadeh, Ehsan Kazemnejad

**Affiliations:** ^1^Reproductive Health Research Center, Department of Obstetrics and Gynecology, Al-zahra Hospital, School of Medicine, Guilan University of Medical Sciences, Rasht, Iran.; ^2^Social Determinants of Health Research Center, Guilan University of Medical Sciences, Rasht, Iran.

**Keywords:** Dyslipidemias, Gestational diabetes, Preeclampsia, Fetal macrosomia.

## Abstract

**Background:**

Concentrations of plasma lipids levels during pregnancy clearly increases. According to some studies, dyslipidemia is effective in the incidence of preeclampsia and insulin resistance. **Objective:** This study aimed to examine the relationship between hyperlipidemia and maternal and neonatal outcomes in pregnant women.

**Materials and Methods:**

This is a cross-sectional study which was conducted on two groups of pregnant women with hyperlipidemia and normal ones to assess maternal and neonatal outcomes. Maternal data including gestational age, mother's age, body mass index, and maternal weight gain during pregnancy, gestational diabetes mellitus, preeclampsia, cholestasis, and delivery method. Also, birth weight and Apgar score were gathered as the neonatal outcomes.

**Results:**

The results showed that the prevalence of abnormal lipid parameters increased with increasing gestational age. In pregnant women with dyslipidemia in combination with increased triglyceride, cholesterol and Low-density lipoprotein, and decreased High-density lipoprotein, the incidence rates of gestational diabetes (p < 0.001), preeclampsia (p < 0.001), cholestasis (p = 0.041), fetal growth retardation (p < 0.001), and macrosomia (p < 0.001) were statistically higher.

**Conclusion:**

Dyslipidemia was associated with some adverse effects of pregnancy and harmful fetal outcomes. Therefore, it seems that adding laboratory assessment of lipid profiles before and during pregnancy can be effective in early diagnosis of dyslipidemia.

## 1. Introduction 

In the first trimester of pregnancy, an increase in serum lipids due to increased lipogenesis and lipolysis suppression can occur. Besides, increased lipolysis and increased levels of fatty acids can be expected at the second trimester of pregnancy. Maternal energy metabolism in the middle of pregnancy is directed toward lipolysis, which leads to an increase in the circulating levels of fatty acids. These changes in lipid metabolism indicate a physiological adaptation of the mother's body that transforms glucose metabolism into lipid metabolism to provide it for fetal development (1). Studies have shown that lipid abnormalities are predictive of insulin resistance (2, 3). Gestational diabetes mellitus (GDM) can affect up to 22% of all pregnancies (1). Gestational diabetes is any type of disorder in glucose during pregnancy which is diagnosed for the first time. The diagnosis is confirmed by administering 75 g glucose and the existence of >= 1 following glucose levels: fasting blood glucose > 92, after 1 hr > 180, and after 2 hr > 153 (1). In women with GDM, physiological changes in insulin and lipid profiles are more severe and may lead to metabolic disorders in this period (4). Few studies have ever assessed the role of lipid profiles in predicting GDM and the results were contradictory (1, 5). Obesity is one of the main risk factors for both insulin resistance and dyslipidemia during pregnancy, which can lead to GDM (6).

Dyslipidemia during pregnancy can also be associated with neonatal outcomes (7). Moreover, Hypertriglyceridemia in obese pregnant women can have negative effects on maternal outcomes and in the long period it can lead to metabolic syndrome in children (8). Disturbances in maternal triglyceride levels during pregnancy and non-esterified fatty acid metabolism are in relation to the fetal overgrowth (9). Recent studies have shown that there was a positive correlation between maternal triglyceride levels and Large for Gestational Age (LGA), which was independent of the maternal glycemic status (10). Also, it has been shown that the concentration of triglyceride was an independent risk factor for macrosomia (11, 12). However, the relationship between maternal triglyceride level and birth weight is still a controversial issue.

Despite studies in this area, it is still unclear how dyslipidemia can be a potential risk factor for insulin resistance during pregnancy. Regarding this issue and also considering that early diagnosis of these disorders can be highly effective in reducing maternal and neonatal complications, and the hospital is the tertiary referral hospital and all high-risk patients were referred from Guilan province; this study aimed to examine the relationship between hyperlipidemia and maternal (GDM, preeclampsia, and cholestasis, etc.) and neonatal outcomes (macrosomia, preterm labor, LGA, small for Gestational Age (SGA), etc.)

## 2. Materials and Methods

### Population and settings

This study is an analytical cross-sectional study which was conducted on the mothers who were eventually referred to the Al-Zahra hospital, Rasht, Iran in 2016-2017. Singleton pregnancies at 28-42 gestational wk (3rd trimester) were included in this study. Mothers with multiple pregnancies, having diabetes type 1 or 2, chromosomal, hereditary metabolic and thyroid diseases before pregnancy, pregnancy with assisted reproductive technologies, who had no glucose tolerance testing during pregnancy, were not included in this study.

Data were collected using a checklist including demographic and laboratory results. Gestational age at the time of delivery was assessed based on the last menstrual period and the ultrasound of the first trimester. Maternal age, weight, and height were recorded and maternal Body mass index (BMI) was calculated based on dividing pre-pregnancy or first-trimester weight (kg) to squared height (m).

The family history of diabetes, the level of education, and the delivery method (natural vaginal delivery or cesarean section) were also recorded. For assessing the fat profile analysis, Hitachi Auto Analyzer, and pars test cholesterol, triglyceride, and high-density lipoprotein (HDL) kits were used. Hyperlipidemia is defined as the presence of one of the following items including serum cholesterol concentration > 200 mg/dL, triglyceride ≥ 150 mg/dL, HDL-C ≤ 45 mg/dL, and low-density lipoprotein (LDL-C) ≥ 130 mg/dL.

After identifying maternal hyperlipidemia, pregnant women were divided into two groups - with and without hyperlipidemia. In order to identify GDM, initial screening by measuring plasma or serum glucose concentration 1 hr after a 50 gr oral glucose load (glucose challenge test (GCT)) and a diagnostic oral glucose tolerance test (OGTT) in the subset of women exceeding the glucose threshold value on GCT. Women were diagnosed as having gestational diabetes if their blood glucose was > 95mg/dl after an overnight fast and was>180,155 and 140 mg/dl after 1, 2, and 3 hr after ingesting a 100gr glucose solution, respectively (13). Preeclampsia was defined as a specific pregnancy-induced disorder characterized with hypertension (systolic blood pressure ≥ 140 mmHg and/or diastolic blood pressure ≥ 90 mmHg) and significant proteinuria (urine protein ≥ 300 mg/24 hr or positive results in random urine protein tests) in previously normotensive women on or after 20 wk gestational age. Cholestasis was determined based on clinical manifestations such as itching and elevated liver enzyme. Biliary obstruction was ruled out by ultrasound. The neonate's birth weight and the first- and fifth-min Apgar scores were measured. Based on the birth weight, those under 2,500 gr were considered low birth weight (LBW), between 2, 500 and 4,000 gr were normal, and those > 4,000 gr were considered microsomes.

### Sample size

According to the formula, 539 pregnant women were assessed. The sample size needed to determine the relationship between hyperlipidemia and some maternal and neonatal outcomes in pregnant women with 95% confidence and 80% power based on the results of Jin *et al*. (14). 

$$1-\alpha =0.95\Rightarrow Z_{1-\alpha /2}=Z_{0.975}=1.96$$

$$1-\beta =0.9\Rightarrow Z_{1-\beta }=Z_{0.9}=0.84$$

Odds ratio = 1.5 preeclampsia P=10%

n=(Z1−α/2+Z1−β)2P(1−P).(ln odds   Ratio )2=(1.96+0.84)20.1.0.9.(Ln1.5)2=539.

### Ethical consideration

This study was approved by the ethics committee of Guilan University of Medical Sciences, Rasht, Iran (Code: IR GUMS.REC.1396.33) and informed consent form was signed by all participants.

### Statistical analysis

Data were analyzed using SPSS (Statistical Package for the Social Sciences, version 21.0, SPSS Inc, Chicago, Illinois, USA). The relationship between hyperlipidemia during pregnancy and GDM and other outcomes were assessed before and after the adjustment of the effects of confounding factors. Chi-square test was used to compare the frequency of maternal outcomes regarding dyslipidemia status. If the test was not valid, Fisher's exact test was used. To compare quantitative normal distributed variables, the independent *t*-test was used and the Mann-Whitney U test was used for non-normal distributed variables. To compare the relationship between hyperlipidemia and maternal and neonatal outcomes with controls, the logistic regression test and the odds ratio were used. P-value < 0.05 indicated statistical significance.

## 3. Results

Totally, 539 pregnant women were participated in this study. The mean age of participants was 30.07 ± 6.15 yr. In terms of educational level, the majority of the women were < diploma (45.1%). The majority of mothers had no history of GDM (85.2%) (Table I). The mean BMI was 27.53 ± 6.11 kg/m${}^{2}$, and the mean weight gain during the pregnancy was 13.26 ± 10.55 kg. The mean of other baseline characteristics are shown in Table II.

According to our results, 364 women (67.5%) had hypertriglyceridemia, 294 (54.5%) had hypercholesterolemia, 152 (28.2%) had abnormal HDL, and 174 (32.3%) had abnormal LDL. Totally, 448 participants (83.1%) had dyslipidemia.

In this study, 31% cases had GDM, 29.9% had preeclampsia, 7.3% had cholestasis in pregnancy, and 16.1% had newborns weighing < 2,500 gr at birth. In terms of delivery, 69.7% had a cesarean section and 30.3% had a vaginal delivery. Chi-square test showed a significant relation between groups regarding the frequency of GDM (p < 0.001), preeclampsia (p < 0.001), cholestasis (p = 0.041), birth weight (p < 0.001), delivery type (p < 0.001), and FGR (p < 0.00). GDM was 35.7% in women with dyslipidemia and 7.7% in healthy women; 34.2% of women with dyslipidemia and 8.8% of healthy women had preeclampsia. Mann-Whitney U test showed that the gestational age at delivery was 37.33 ± 2.13 in patients and 38.04 ± 1.85 in normal people, and this difference was statistically significant (p < 0.0001). Birth weight in the dyslipidemia group was 3151.41 ± 743.76 and in the non-affected group was 3195.27 ± 440.83, which was not statistically significant (p = 0.861). Apgar scores were statistically significant in the first minute (p < 0.001) and in the fifth minute (p < 0.001), and the newborns of dyslipidemic mothers had lower Apgar scores than the infants of healthy mothers (Table III).

There was no significant relation between early and late preterm labor (p = 0.006). Comparing patients with early preterm labor showed a significant relation in terms of hypertriglyceridemia (p = 0.027), hypercholesterolemia (p = 0.002), and abnormal LDL (p = 0.0007). Furthermore, late preterm labor showed a significant relation in terms of hypertriglyceridemia (p = 0.001), hypercholesterolemia (p = 0.001), and abnormal LDL (p = 0.001). The rate of macrosomia was different in terms of hypertriglyceridemia (p = 0.001), hypercholesterolemia (p = 0.001), and hyperlipidemia (p = 0.001) (Table IV).

Multiple logistic regression analysis was used to analyze the relationship between dyslipidemia and GDM after adjusting the effects of individual variables and disease records. Dyslipidemia increased 4.1 folds the chance of a pregnancy-induced GDM (Odds ratio= 4.1 95% CI OR: 1.8-9.5). In addition to dyslipidemia, the maternal age (p = 0.001, OR: 1.1), maternal education (p = 0.01, OR = 0.679), BMI (p = 0.001, OR = 1.1), and also the history of GDM in the previous pregnancy (p = 0.011, OR = 5.6) had a significant relationship with GDM. Among these variables, only maternal education had a protective effect (protecting odds ratio < 1) and others were risk factors. Results showed that by modulating the effects of demographic and confounding variables, dyslipidemia was considered as a factor associated with preeclampsia (p = 0.0001) (odd = 4.1 95% CI OR = 1.9-8.8). In addition to hyperlipidemia, the maternal age (p = 0.002, OR = 1.05) and BMI (p = 0.005, OR = 1.05) had a risk factor effect on preeclampsia. Results showed that dyslipidemia has not been considered as a factor associated with the symptoms of cholestasis in pregnancy by modulating the effects of demographic and confounding variables (p = 0.07) (odd = 3.7 95% CI OR = 0.889-15.9).

However, the history of diabetes (p = 0.02, OR = 3.9) had a risk factor effect for the symptoms of cholestasis. Based on the results of the final model, logistic regression showed dyslipidemia as a factor associated with positive FGR by modulating the effects of demographic and confounding variables (p = 0.003) (odd = 4.8 95% CI OR = 1.69-13.39). Also, educational level (p = 0.05, OR = 1.4) had a risk factor effect on positive FGR (Borderline Significance). Based on the results of the final model, logistic regression of dyslipidemia has not been considered as a factor related to cesarean delivery by modulating the effects of demographic and confounding variables (p = 0.081) (odd = 1.56 95% CI OR = 0.946-2.58). However, maternal age (p = 0.001, OR = 1.12), BMI (p = 0.009, OR = 1.05) had a risk factor effect on cesarean delivery.

**Table 1:** Frequency distribution of demographic characteristics (n = 539)


**Variables**		**N (%)**
Maternal age (yr)		
	< 20	40 (7.4)
	21-25	92 (17.1)
	26-30	129 (23.9)
	31-35	167 (31.0)
	> 35	111 (20.6)
History of GDM in previous pregnancies		80 (14.8)
Diabetes inheritance in mother or father		156 (29.1)
BMI		
	< 18.5	24 (4.5)
	18.5-24.99	161 (29.9)
	25-29.99	202 (37.5)
	≥ 30	152 (28.2)
LDL		
	> 150	174 (32.3)
	< 150	365 (67.7)
HDL		
	> 50	152 (28.2)
	< 50	387 (71.8)
GDM: Gestational diabetes mellitus; BMI: Body mass index; HDL: High-density lipoprotein; LDL: Low-density lipoprotein		

**Table 2:** The mean of baseline characteristics


**Variables**	
Maternal weight before or during the first trimester	71.14 ± 14.63
Height	161.00 ± 6.86
Triglyceride	218.61 ± 106.52
Cholesterol	217.50 ± 60.12
HDL	53.15 ± 11.72
LDL	123.21 ± 48.31

**Table 3:** Distribution of pregnancy outcomes in women with and without hyperlipidemia


	**Hyperlipidemia**		**P-value**
	**Positive**	**Negative**	
BMI				
	< 18.5	15 (3.3)	9 (9.9)	
	18.5-24.99	126 (28.1)	35 (38.5)	
	25-29.99	166 (37.1)	36 (39.6)	
	≥ 30	141 (31.5)	11 (12.1)	0.001*
GDM		160 (35.7)	7 (7.7)		0.001*
Preeclampsia		153 (34.2)	8 (8.8)		0.001*
Cholestasis		37 (8.3)	2 (2.2)		0.041*
Birth weight				
	< 2500	79 (17.6)	8 (8.8)	
	2500-4000	319 (71.2)	83 (91.2)	
	> 4000	50 (11.2)	0 (0.0)	0.001*
Delivery type				
	NVD	121 (27.0)	42 (46.7)		
	C/S	327 (73.0)	48 (53.3)	0.001*
FGR		80 (17.9)	4 (4.4)		0.001*
1 min Apgar score				
	< 7	42 (9.4)	2 (2.2)	
	≥ 7	406 (90.6)	89 (97.8)	0.023*
5 min Apgar score				
	< 7	5 (1.1)	0 (0.0)	
	≥ 7	443 (98.9)	91 (100.0)	0.311*
Gestational age (wk)		37.35 ± 2.13	38.04 ± 1.85		0001**
Birth weight (gr)		3151.41 ± 743.76	3195.27 ± 440.83	0.861**
1 min Apgar score		7.83 ± 1.04	8.26 ± 0.85	0.001**
5 min Apgar score		8.94 ± 0.84	9.30 ± 0.61	0.001**
Data presented as n (%)				
GDM: Gestational diabetes mellitus; NVD: Normal vaginal delivery C/S: Cesarean section; FGR: Fetal uterine growth retardation				
*Chi-square test; **Mann-Whitney U test: Data presented as mean ± SD				

**Table 4:** Frequency distribution of preterm labor and macrosomia based on maternal lipid profile


	**Early preterm labor**		**Late preterm labor**		**Macrosomia n (%)**	
	**GA ≥ 34 week**	**GA < 34 week**	**GA ≥ 37 week**	**GA < 37 week**	
			**No**	**Yes**
Hypertriglyceridemia		339 (93.1)	25 (6.9)	269 (73.9)	95 (26.1)	47 (12.9)	317(87.1)
	p-value	0.027**		0.001*		0.001**	
Hypercholesterolemia		270 (91.8)	24 (8.2)	215 (73.1)	79 (26.9)	251 (85.4)	43 (14.6)
	p-value	0.002*		0.001*		0.001*	
Abnormal HDL		144 (94.7)	8 (5.3)	124 (81.6)	28 (18.4)	143 (94.1)	9 (5.9)
	p-value	0.947**		0.246*		0.092	
Abnormal LDL		158 (90.8)	16 (9.2)	119 (68.4)	55 (31.6)	152 (87.4)	22 (12.6)
	p-value	0.007*		0.001*		0.063*	
Hyperlipidemia		421 (94.0)	27 (6.0)	344 (76.8)	104 (23.2)	8398 (8.8)	50 (11.2)
	p-value	0.140**		0.06		0.001**	
Data presented as n(%)						
*Chi-square test; **Fisher's Exact test						
HDL: High-density lipoprotein; LDL: Low-density lipoprotein; GA: Gestational age						

## 4. Discussion

In this study, the relationship between hyperlipidemia and GDM and other maternal and child-related outcomes showed that the higher the gestational age, the greater the incidence of lipid parameters. There was a significant relation between dyslipidemia with the incidence of GDM, preeclampsia, cholestasis in pregnancy, and macrosomia. Jin and colleagues mentioned that the mean age of mothers and the mean weight gain during pregnancy were similar to the current study, and P-BMI and educational level were different (14). Also, in the study of Li and others, the mean age of mothers and P-BMI were similar to this study and the level of education and family history of diabetes were different (3). Given that the mean BMI of the current population was within the overweight range (25-29.9), the overall weight gain in this group was higher than the expected weight gain (7.5-11). Lower levels of education can lead to high levels of obesity and higher BMI by creating cultural, socioeconomic, and lifestyle differences, and excessive consumption of high-calorie foods. According to the classification and lipid profile, 83.1% (448) had dyslipidemia. Two constant manifestations of lipid metabolism changes in a pregnant mother that occurs normally during pregnancy are lipid accumulation in the maternal tissues and the occurrence of hyperlipidemia. Fat accumulation at the beginning of pregnancy with hyperphagia can also increase lipogenesis (15). Hyperlipidemia during pregnancy is a physiological phenomenon which occurs as a result of increased insulin resistance and the synthesis of lipoproteins and lipolysis in adipose tissue, in order to provide fats as a source of energy for the development of the fetus (16). The majority of pregnant women commonly show an increase in triglycerides in the third trimester, an increase in HDL in the second half of pregnancy, and a progression of IDL and LDL during pregnancy (1). In the study of Jin and colleagues, the presence of GDM (7.6%), preeclampsia (1.5%), and SGA (71.3%) were different from this study, and the occurrence of macrosomia, gestational age, and birth weight were consistent.(14) Schaefer and co-worker found higher frequency rates of GDM (76%) and macrosomia (18.9%) (8). In the study of Wang and co-worker, lower frequency of GDM (20.2%) was noted (17). However, similar gestational age and birth weight were noted by Li and co-worker (11). Considering that this study was conducted at the referral hospital of the capital of Guilan province, different results compared with previous investigations regarding GDM and preeclampsia may be caused by referring high-risk pregnancies to the center. Although, previous investigations did not assess perinatal care, delivery type, and Apgar scores, in this study, 98.5% of the patients had perinatal care that indicated appropriate culture and awareness. According to the expectations, cesarean section was more than NVD. One significant reason was the repeated cesarean delivery and the lack of vaginal birth after cesarean section (18). Also, an increased inability to initiate labor and subsequently induction by oxytocin and cesarean section are more common in people with abnormal weight (19), and it decreases the likelihood of successful vaginal birth after cesarean section in women (18). The frequency of GDM and preeclampsia were statistically significant in women with dyslipidemia and healthy ones. Also, higher frequencies were noted in terms of cholestasis, LBW, and cesarean section in women with dyslipidemia. In the study of Jin and colleagues, there was a significant relationship between dyslipidemia and an increased risk of GDM, preeclampsia, and macrosomia (14). In the study of Liu and colleagues and Wang and colleagues, there was a significant relationship between dyslipidemia and GDM (11, 17). In pregnant women with GDM, the incidence of insulin resistance syndrome (metabolic syndrome) was also high. Pregnant women with diabetes mellitus had higher rates of central obesity, glucose levels, insulin, triglycerides, total cholesterol, and LDL compared to the control group. It was also associated with GDM with early onset of atherosclerosis (20). Endothelial dysfunction is an early onset sign of atherosclerosis which usually occurs shortly after the delivery in women with the history of GDM. Therefore, generally, women with diabetes mellitus are not only at risk for type 2 diabetes but also at an increased risk of cardiovascular complications associated with abnormal serum fat and hypertension and abdominal obesity (21). The association of dyslipidemia with cholestasis can be explained by the theoretical description provided on reducing the activity of FXR and TGR5 acid receptors, although the actual mechanism needs to be further investigated (14). Considering hyperlipidemia as an oxidative suppressant stimulus and the inflammation associated with the incidence of pregnancy complications and its harmful effects such as preeclampsia, diabetes, and LGA (22), the occurrence of preterm labor can be justified. Although the relationship between maternal dyslipidemia and preterm delivery is not consistent and this relationship has not been reported in any systematic reviews and meta-analysis.

### Limitation

This study had some limitations but the investigators considered them to eliminate as far as possible. Weight gain before and during pregnancy was self-reported and may be biased. In this study, other confounding factors were not assessed, for example, receiving adequate or excessive diet during pregnancy and sufficient physical activity during pregnancy, which may have an effect on the incidence of dyslipidemia. Therefore, it seems that more studies are needed to investigate the relationship between the mother's lifestyle and the incidence of dyslipidemia during pregnancy.

## 5. Conclusion

According to the results of the present study, it seems that adding laboratory assessment of lipid profiles before and during pregnancy can be effective in early diagnosis of dyslipidemia. Considering that most drugs used to treat hyperlipidemia during pregnancy are in the C or X groups, treatment can be related to changing the lifestyle by increased physical activity, controlling calorie intake, and timely treatment of disorders such as diabetes and hypertension.

##  Conflict of Interest

The authors declare that they have no conflict of interests.
